# Psychrosphaera algicola sp. nov. and Paraglaciecola algarum sp. nov., and reclassification of Pseudoalteromonas elyakovii, Pseudoalteromonas flavipulchra, and Pseudoalteromonas profundi as later heterotypic synonyms of P. distincta, P. maricaloris, and P. gelatinilytica

**DOI:** 10.1099/ijsem.0.006491

**Published:** 2024-08-14

**Authors:** Hülya Bayburt, Byeong Jun Choi, Jeong Min Kim, Ju Hye Baek, Che Ok Jeon

**Affiliations:** 1 Department of Life Science, Chung-Ang University, Seoul 06974, Republic of Korea

**Keywords:** marine alga, new taxa, *Paraglaciecola algarum*, *Pseudomonadota*, *Psychrosphaera algicola*

## Abstract

Two Gram-negative, obligately aerobic, rod-shaped bacteria, strains G1-22^T^ and G1-23^T^, were isolated from the phycosphere of a marine brown alga. Both strains exhibited catalase- and oxidase-positive activities. Strain G1-22^T^ displayed optimal growth at 25 °C, pH 8.0, and 2.0–3.0% (w/v) NaCl, while strain G1-23^T^ exhibited optimal growth at 25 °C, pH 8.0, and 4.0% NaCl. Ubiquinone-8 was identified as the sole isoprenoid quinone in both strains. As major fatty acids (> 5%), strain G1-22^T^ contained C_16 : 0_, summed feature 3 (C_16 : 1_ *ω*7*c* and/or C_16 : 1_ *ω*6*c*), summed feature 8 (C_18 : 1_ *ω*7*c* and/or C_18 : 1_ *ω*6*c*), C_12 : 1_ 3-OH, and C_10 : 0_ 3-OH, while strain G1-23^T^ contained C_16 : 0_, summed feature 3 (C_16 : 1_ *ω*7*c* and/or C_16 : 1_ *ω*6*c*), and C_14 : 0_. Phosphatidylethanolamine, phosphatidylglycerol, and diphosphatidylglycerol were major polar lipids in both strains. Strains G1-22^T^ and G1-23^T^ had DNA G+C contents of 40.2 and 38.9 mol%, respectively. Phylogenetic analyses based on 16S rRNA and genome sequences revealed that strains G1-22^T^ and G1-23^T^ formed distinct phylogenetic lineages within the genera *Psychrosphaera* and *Paraglaciecola*, respectively. Strain G1-22^T^ showed closest relatedness to *Psychrosphaera ytuae* MTZ26^T^ with 97.8% 16S rRNA gene sequence similarity, 70.2% average nucleotide identity (ANI), and a 21.5% digital DNA–DNA hybridization (dDDH) value, while strain G1-23^T^ was most closely related to *Paraglaciecola aquimarina* KCTC 32108^T^ with 95.6% 16S rRNA gene sequence similarity, 74.6% ANI, and a 20.1% dDDH value. Based on phenotypic and molecular characteristics, strains G1-22^T^ and G1-23^T^ are proposed to represent two novel species, namely *Psychrosphaera algicola* sp. nov. (type strain G1-22^T^=KACC 22486^T^=JCM 34971^T^) and *Paraglaciecola algarum* sp. nov. (type strain G1-23^T^=KACC 22490^T^=JCM 34972^T^), respectively. Additionally, based on the comparison of whole genome sequences, it is proposed that *Pseudoalteromonas elyakovii*, *Pseudoalteromonas flavipulchra*, and *Pseudoalteromonas profundi* are reclassified as later heterotypic synonyms of *Pseudoalteromonas distincta*, *Pseudoalteromonas maricaloris*, and *Pseudoalteromonas gelatinilytica*, respectively.

## Introduction

The genus *Psychrosphaera*, classified within the family *Pseudoalteromonadaceae* of the phylum *Pseudomonadota*, was first proposed in 2010, with *Psychrosphaera saromensis* as the type species, isolated from Lake Saroma [1]. As of May 2024, this genus comprises five validly published species (https://lpsn.dsmz.de/genus/psychrosphaera), primarily isolated from marine environments, including coastal lagoon [1], marine invertebrate [2], seawater [2,3], and marine sediment [3,4]. Cells of the genus *Psychrosphaera* are characterized by Gram-stain-negative, non-pigmented, obligately aerobic, motile rods, or coccoids with a single polar flagellum, exhibiting positive reactions for oxidase and catalase. Furthermore, members of this genus predominantly contain ubiquinone-8 (Q-8) as the respiratory quinone and phosphatidylethanolamine (PE), phosphatidylglycerol (PG), and diphosphatidylglycerol (DPG) as major polar lipids, with genomic DNA G+C contents ranging from 38.7 to 49.5% [1,4].

The genus *Paraglaciecola*, classified within the family *Alteromonadaceae* of the phylum *Pseudomonadota*, was initially established by reclassifying members from the genus *Glaciecola* into a distinct genus, with *Paraglaciecola mesophila* as the type species [5]. As of May 2024, the genus *Paraglaciecola* encompasses 11 validly published species (https://lpsn.dsmz.de/genus/paraglaciecola), isolated from diverse marine environments, including marine alga [6], seawater [7,8], the Arctic [9,11], seaweed [12], and marine sediment [13]. Cells of the genus *Paraglaciecola* are Gram-stain-negative, obligate aerobes, and are motile with an ovoid, rod, or slightly curved shape, featuring a single polar flagellum. They also test positive for oxidase and catalase. Additionally, members of this genus predominantly possess Q-8 as the respiratory quinone and PE, PG, and DPG as major polar lipids, with genomic DNA G+C contents ranging from 38.3 to 47.0% [5,13].

Our studies investigating marine algae–bacteria interactions have led to the isolation of numerous novel bacteria from the phycosphere of marine algae [14,17]. In this study, we isolated two potentially novel species within the genera *Psychrosphaera* and *Paraglaciecola* from a marine brown alga and characterized their taxonomic characteristics using a polyphasic approach. In addition, it is proposed that *Pseudoalteromonas elyakovii* (Ivanova *et al*. 1987) Sawabe *et al*. 2000, *Pseudoalteromonas flavipulchra* Ivanova *et al*. 2002, and *Pseudoalteromonas profundi* Zhang *et al*. 2016 should be reclassified as later heterotypic synonyms of *Pseudoalteromonas distincta* (Romanenko *et al*. 1995) Ivanova *et al*. 2000, *Pseudoalteromonas maricaloris* Ivanova *et al*. 2002, and *Pseudoalteromonas gelatinilytica* Yan *et al*. 2016, respectively, based on a comparison of their genome sequences.

## Strain isolation

Strains G1-22^T^ and G1-23^T^ were isolated from the phycosphere of a marine brown alga, specifically a *Sargassum* species, collected from the sea coastal area of Gonghyeonjin (38° 21′ 21″ N, 128° 30′ 45″ E) in Gangwon province, Republic of Korea, in June 2021. In brief, a collected brown alga was thoroughly washed using artificial seawater (ASW; 20.0 g NaCl, 2.9 g MgSO_4_, 4.5 g MgCl_2_·6H_2_O, 0.6 g KCl, 1.8 g CaCl_2_·2H_2_O per litre) through mechanical vortexing. Subsequently, the washed alga was homogenized mechanically using an Ultra-Turrax homogenizer (IKA), followed by serial dilution in ASW. Aliquots of 100 µl from each dilution were spread on marine agar (MA; MBcell) and incubated aerobically at 25 °C for 7 days. Colonies grown on MA were subjected to PCR amplification of the 16S rRNA gene using the universal primers 27F (5′-AGAGTTTGATCMTGGCTCAG-3′) and 1492R (5′-TACGGYTACCTTGTTACGACTT-3′) [16] after boiling in 100 µl of 5% (w/v) Chelex 100 solution (Bio-Rad) for 10 min. The resulting PCR products were double-digested with restriction enzymes *Hae*III and *Hha*I and analysed via 2% (w/v) agarose gel electrophoresis. Distinctive fragment patterns were sequenced using the universal primer 340F (5′-CCTACGGGAGGCAGCAG-3′) [17] at Macrogen (Seoul, Republic of Korea), and the resulting sequences were compared with those of all validly published type species available in the EzBioCloud server (www.ezbiocloud.net/identify) [18].

Two potentially novel strains within the genera *Psychrosphaera* and *Paraglaciecola*, designated as strains G1-22^T^ and G1-23^T^, were identified from this comparison and selected for further taxonomic characterization. These isolates were routinely cultured on MA for 2 days at 25 °C and preserved long-term at −80 °C in marine broth (MB; MBcell) supplemented with 15% (v/v) glycerol.

## Phylogeny based on 16S rRNA gene sequences

The 16S rRNA gene amplicons from strains G1-22^T^ and G1-23^T^, generated using the 27F and 1492R primers, were subsequently sequenced with universal primers 518R (5′-ATTACCGCGGCTGCTGG-3′) and 805F (5′-GATTAGATACCCTGGTAGTC-3′) [16]. Sequences obtained from sequencing using 340F, 518R, and 805F primers were assembled to yield nearly complete 16S rRNA gene sequences for strains G1-22^T^ (1478 nucleotides) and G1-23^T^ (1442 nucleotides). The 16S rRNA gene sequence similarities between strains G1-22^T^ and G1-23^T^ and their closely related type strains were determined using the nucleotide similarity search program on the EzBioCloud server [18]. Subsequently, the 16S rRNA gene sequences of strains G1-22^T^ and G1-23^T^, along with those of their closely related type strains, were aligned, and phylogenetic trees with bootstrap values (1000 replications) were reconstructed using the maximum-likelihood (ML), neighbour-joining (NJ), and maximum-parsimony (MP) algorithms in mega11 software [19]. For the reconstruction of NJ, ML, and MP trees, the Kimura two-parameter model, nearest-neighbor-interchange heuristic search method, and complete deletion options were used, respectively.

Comparative analysis of 16S rRNA gene sequences revealed that strain G1-22^T^ exhibited the highest sequence similarities of 97.8, 97.0, and 97.0% to *Psychrosphaera ytuae* MTZ26^T^, *Psychrosphaera saromensis* SA4-48^T^, and *Psychrosphaera aquimarina* SW33^T^, respectively, while strain G1-23^T^ showed the highest sequence similarities of 95.6, 95.4, and 94.8% with * Paraglaciecola aquimarina* GGW-M5^T^, *Paraglaciecola marina* D3211^T^, and *Paraglaciecola aestuariivivens* JDTF-33^T^, respectively. The 16S rRNA gene sequence similarity between strains G1-22^T^ and G1-23^T^ was only 86.4%.

**Fig. 1.**
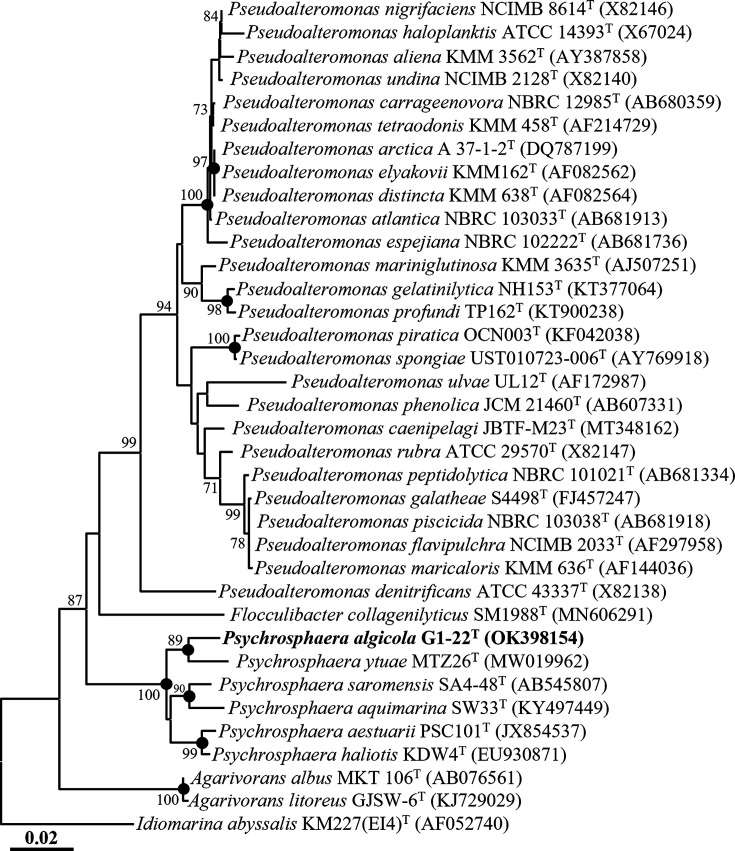
Neighbour-joining tree based on 16S rRNA gene sequences showing the phylogenetic relationships between strain G1-22^T^ and their closely related taxa. Filled circles (●) designate the nodes that were also redeemed in the maximum-likelihood and maximum-parsimony trees. Bootstrap values greater than 70% based on 1000 replicates are indicated at branching points. *Idiomarina abyssalis* KM227(EI4)^T^ (AF052740) was used as the outgroup for strain G1-22^T^. Scale bar, 0.01 substitution per nucleotide.

**Fig. 2.**
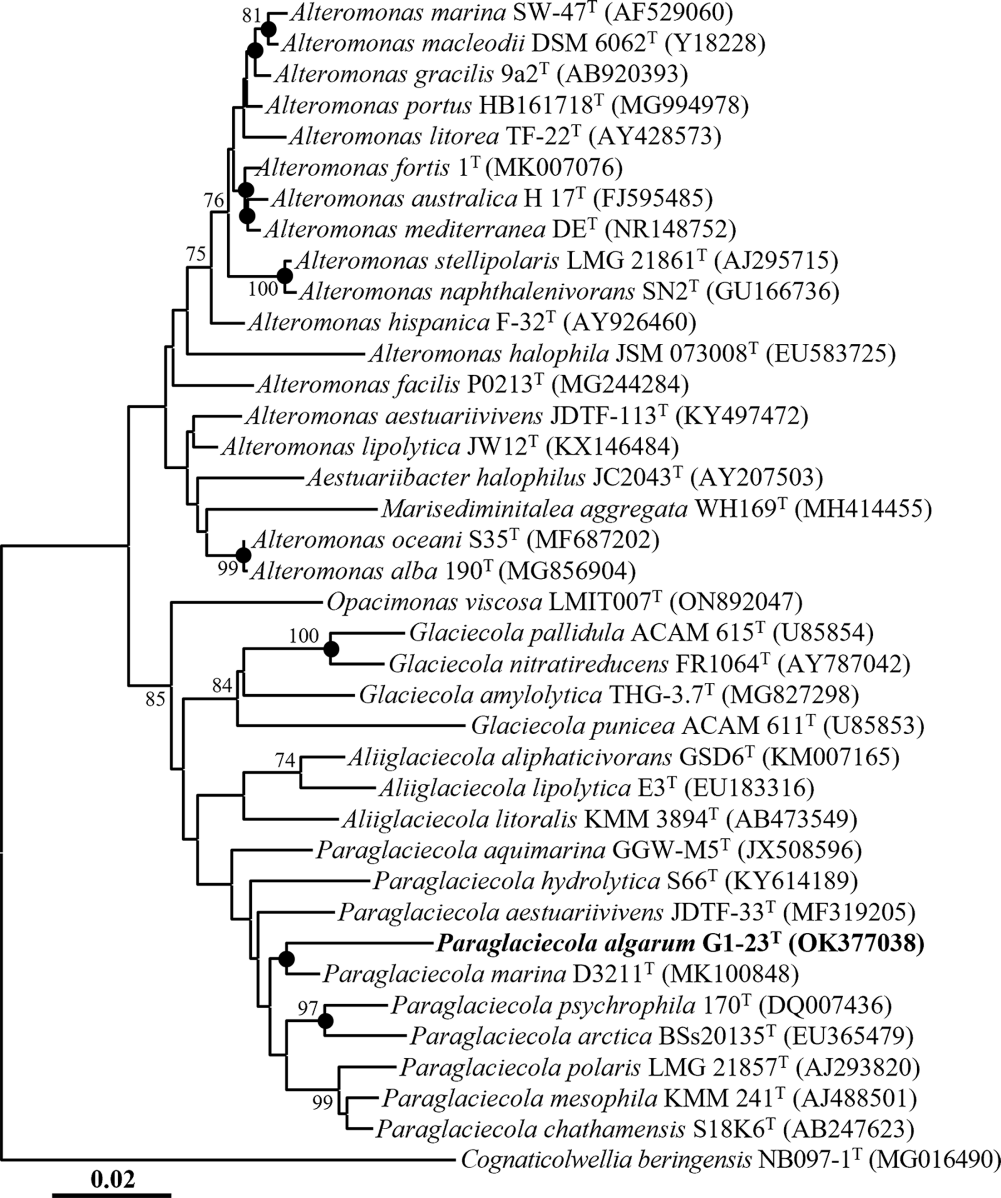
Neighbour-joining tree based on 16S rRNA gene sequences showing the phylogenetic relationships between strain G1-23^T^ and their closely related taxa. Filled circles (●) designate the nodes that were also redeemed in the maximum-likelihood and maximum-parsimony trees. Bootstrap values greater than 70% based on 1000 replicates are indicated at branching points. *Cognaticolwellia beringensis* NB097-1^T^ (MG016490) was used as the outgroup for strain G1-23^T^. Scale bar, 0.02 substitution per nucleotide.

In the phylogenetic tree reconstructed using the NJ algorithm, strain G1-22^T^ formed a phyletic lineage with *Psychrosphaera ytuae* MTZ26^T^ with an 89% bootstrap value within the genus *Psychrosphaera* (Fig. 1), while strain G1-23^T^ formed a phyletic lineage with *Paraglaciecola marina* D3211^T^ within the genus *Paraglaciecola* (Fig. 2). Phylogenetic trees reconstructed using the ML and MP algorithms consistently indicated that strains G1-22^T^ and G1-23^T^ formed clusters with *Psychrosphaera ytuae* MTZ26^T^ and *Paraglaciecola marina* D3211^T^ within their respective genera (Fig. S1, available in the online version of this article). These 16S rRNA gene sequence-based analyses suggest that strains G1-22^T^ and G1-23^T^ likely represent novel species members of the genera *Psychrosphaera* and *Paraglaciecola*, respectively.

Based on the results from 16S rRNA gene sequence-based analyses, *Psychrosphaera ytuae* JCM 34321^T^, *Psychrosphaera saromensis* KCTC 23240^T^, *Psychrosphaera aquimarina* KCTC 52743^T^, *Paraglaciecola aquimarina* KCTC 32108^T^ and *Paraglaciecola marina* KCTC 72122^T^, obtained from their respective culture collections, were selected as reference strains for comparative genomic characteristics, phenotypic properties, and fatty acid compositions with strains G1-22^T^ and G1-23^T^.

## Whole genome sequencing and phylogeny based on genome sequences

The genomic DNA of strains G1-22^T^ and G1-23^T^, as well as reference strains *Psychrosphaera aquimarina* KCTC 52743^T^ and *Paraglaciecola aquimarina* KCTC 32108^T^, was extracted from cells cultured in MB using the Wizard Genomic DNA purification kit from Promega, following the manufacturer’s instructions. The genomic DNA of strains G1-22^T^, KCTC 52743^T^, and KCTC 32108^T^ were subsequently sequenced on an Oxford Nanopore MinION sequencer (ONT), and the resulting sequencing reads were subjected to *de novo* assembly using Flye (version 2.9.1) [20]. The genomic DNA of strain G1-23^T^ was sequenced using the Illumina HiSeq X platform with 151 bp paired-end reads at Macrogen, and the resulting sequencing reads were subjected to *de novo* assembly using SPAdes (version 3.12.0) [21]. The quality of the assembled genomes was assessed based on their completeness and contamination rates using CheckM2 software (version 1.0.2) [22].

The *de novo* assembly of the genome sequencing data yielded complete genomes comprising three contigs, consisting of a 4692 kb chromosome, a 2.1 kb plasmid, and a 1.9 kb plasmid, for strain G1-22^T^ and two contigs, consisting of a 4736 kb chromosome and a 153.7 kb plasmid, for *Paraglaciecola aquimarina* KCTC 32108^T^ (Table 1). Conversely, it resulted in draft genomes comprising 52 and 10 contigs for strain G1-23^T^ and *Psychrosphaera aquimarina* KCTC 52743^T^, with the N50 values of 366.7 kb and 1047 kb, respectively. The completeness and contamination rates of the assembled genomes for strains G1-22^T^, G1-23^T^, KCTC 52743^T^, and KCTC 32108^T^ were predicted to be 93.4, 99.8, 98.5, and 98.4% for completeness, and 5.9, 0.4, 3.5, and 5.0% for contamination rate, respectively, meeting the criteria for generally high-quality genomes (≥ 90% completeness; ≤ 10% contamination rate) [22].

**Table 1.** General genomic characteristics of strains G1-22^T^ and G1-23^T^, as well as closely related type strains of the genera *Psychrosphaera* and *Paraglaciecola* Strains: 1, G1-22^T^ (JAQOMS000000000); 2, *Psychrosphaera saromensis* KCTC 23240^T^ (BMYG00000000); 3, *Psychrosphaera aquimarina* KCTC 52743^T^ (JAWCUA000000000); 4, *Psychrosphaera ytuae* MTZ26^T^ (CP072110); 5, strain G1-23^T^ (JAKGAS000000000); 6, *Paraglaciecola marina* D3211^T^ (SAIS00000000); 7, *Paraglaciecola aquimarina* KCTC 32108^T^ (JAWDIO000000000). The genomes of strains G1-22^T^ and G1-23^T^, *P. sychrosphaera aquimarina* KCTC 52743^T^, and *Paraglaciecola aquimarina* KCTC 32108^T^ were sequenced in this study.

Charateristics*	1	2	3	4	5	6	7
Genome status (no. of contigs)†	C (3)	D (24)	D (10)	C (1)	D (52)	D (75)	C (2)
Genome size (kb)	4695	3605	3870	3331	4818	4638	4893
N50 (kb)	4691	305.7	1047	3331	366.7	208.4	4739
G+C content (mol%)	40.2	37.7	37.8	42.2	38.9	39.4	41.1
No. of total genes	5161	3261	3602	2942	4123	4171	4695
No. of protein-coding genes	3995	3162	3414	2837	4032	4086	3962
No. of total RNA	93	78	79	94	59	59	73
No. of tRNA	74	63	65	70	51	52	57
No. of rRNA	15	11	9	19	4	3	12
No. of non-coding RNA	4	4	5	5	4	4	4
No. of pseudogenes	1073	21	109	11	32	26	660
No. of total CAZy† genes	166	48	81	64	199	105	191
Glycoside hydrolases	99	18	38	25	98	34	122
Glycosyltransferases	22	15	16	18	32	33	31
Polysaccharide lyases	7	2	7	1	24	10	15
Carbohydrate esterases	22	8	13	12	19	18	16
Auxiliary activities	3	2	3	5	10	6	2
Carbohydrate-binding modules	13	3	4	3	16	4	5

†*The bioinformatic analysis of the genomes was carried out using the NCBI Prokaryotic Genome Annotation Pipeline (www.ncbi.nlm.nih.gov/genome/annotation_prok/).

‡†C, complete; D, draft; CAZy, carbohydrate-active enzyme.

A genome-based phylogenomic analysis was conducted on strains G1-22^T^ and G1-23^T^, alongside closely related type strains, using the Genome Taxonomy Database Toolkit (GTDB-Tk), based on the concatenated protein sequences of 120 ubiquitous single-copy marker genes (bac120 marker set) [23]. The alignment of these concatenated protein sequences and subsequent phylogenomic ML tree reconstruction, including bootstrap values (derived from 1000 replications), were performed using mega11 software. Average nucleotide identity (ANI) and digital DNA–DNA hybridization (dDDH) values among the genomes of strains G1-22^T^ and G1-23^T^ and their closely related type strains were calculated using the Orthologous ANI Tool software available on the EzBioCloud server (www.ezbiocloud.net/tools/orthoani) [24] and the online Genome-to-Genome Distance Calculator version 3.0 (https://ggdc.dsmz.de/ggdc.php) with formula 2 [25], respectively.

**Fig. 3.**
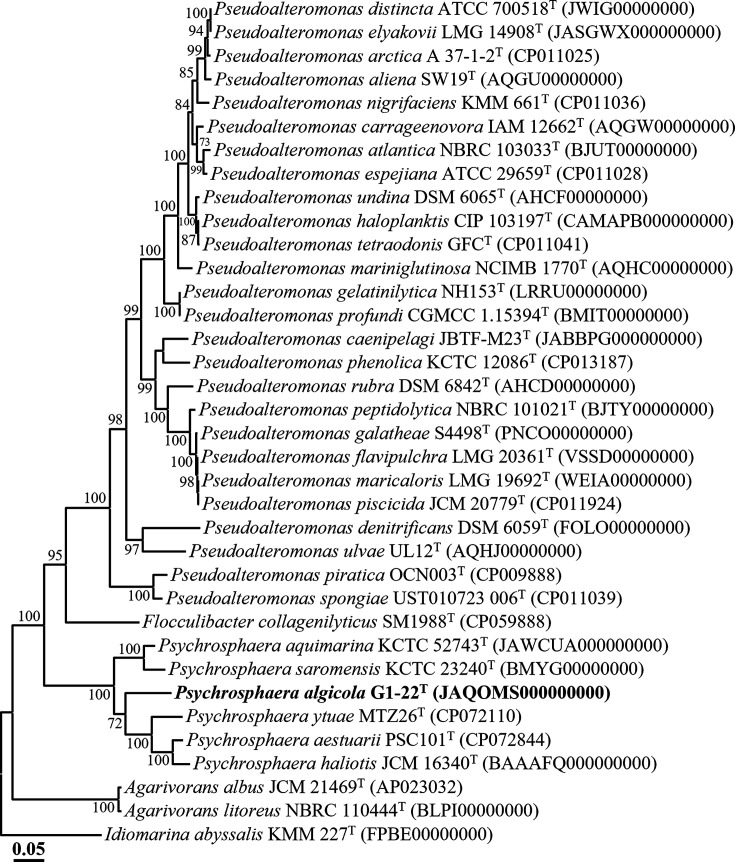
Phylogenomic tree showing the phylogenetic relationships of strain G1-22^T^ and their closely related taxa, based on the concatenation of 120-bacterial protein marker set (bac120 marker set) of GTDB-Tk. Only bootstrap values exceeding 70% are indicated on the nodes as percentages from 1000 replicates. *Idiomarina abyssalis* KMM 227^T^ (FPBE00000000) was used as the outgroup for the strain G1-22^T^. Scale bar, 0.05 substitution per amino acid.

**Fig. 4.**
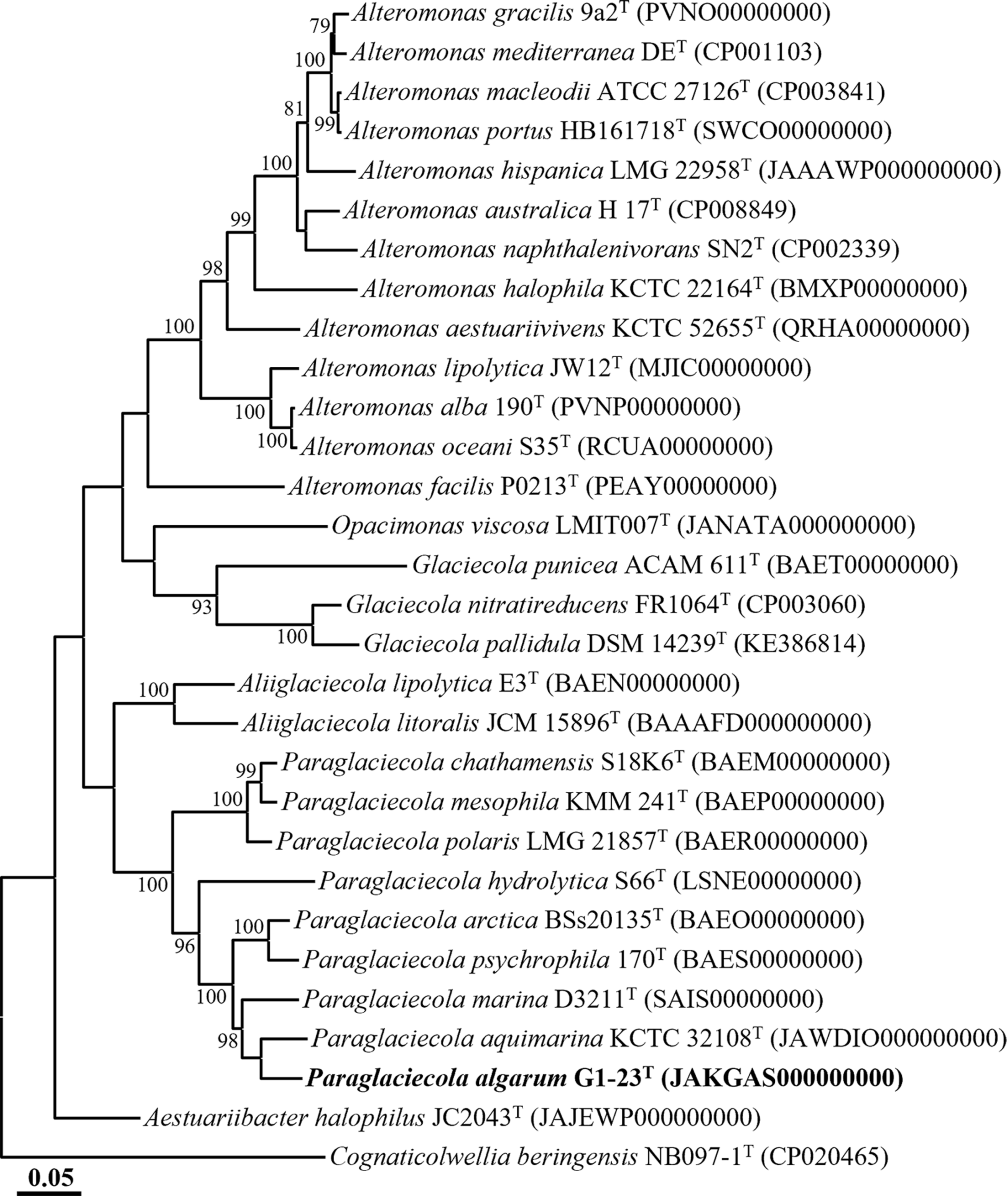
Phylogenomic tree showing the phylogenetic relationships of strain G1-23^T^ and their closely related taxa, based on the concatenation of 120-bacterial protein marker set (bac120 marker set) of GTDB-Tk. Only bootstrap values exceeding 70 % are indicated on the nodes as percentages from 1000 replicates. *Cognaticolwellia beringensis* NB097-1^T^ (CP020465) were used as the outgroup for the strain G1-23^T^. Scale bar, 0.05 substitution per amino acid.

The genome-based phylogenomic trees revealed that strains G1-22^T^ and G1-23^T^ formed distinct lineages within the genera *Psychrosphaera* (Fig. 3) and *Paraglaciecola* (Fig. 4), respectively, which further corroborates the conclusion drawn from the analyses based on 16S rRNA gene sequences, confirming that strains G1-22^T^ and G1-23^T^ indeed belong to the genera *Psychrosphaera* and *Paraglaciecola*, respectively. The genome relatedness between strain G1-22^T^ and its closely related type strains *Psychrosphaera ytuae* MTZ26^T^, *Psychrosphaera saromensis* KCTC 23240^T^, and *Psychrosphaera aquimarina* KCTC 52743^T^ were 70.2, 71.3, and 71.2% for ANI, and 21.5, 21.3, and 20.4% for dDDH, respectively (Table 2). Meanwhile, the genome relatedness between strain G1-23^T^ and its closely related type strains *Paraglaciecola aquimarina* KCTC 32108^T^ and *Paraglaciecola marina* D3211^T^ were 74.6 and 72.4% for ANI, and 20.1 and 19.9 % for dDDH, respectively (Table 3). These ANI and dDDH values were notably below the thresholds (ANI, ~95–96%; dDDH, 70%) for prokaryotic species delineation [26]. In conclusion, the results from the phylogenomic analysis and genome relatedness assessments strongly support the conclusion that strains G1-22^T^ and G1-23^T^ represent novel species within the genera *Psychrosphaera* and *Paraglaciecola*, respectively.

**Table 2.** Genome relatedness between strain G1-22^T^ and closely related type strains of the genus *Psychrosphaera* Strains: 1, G1-22^T^ (JAQOMS000000000); 2, *P. saromensis* KCTC 23240^T^ (BMYG00000000); 3, *P. aquimarina* KCTC 52743^T^ (JAWCUA000000000); 4, *P. ytuae* MTZ26^T^ (CP072110); 5, *P. aestuarii* PSC101^T^ (CP072844); 6, *P. haliotis* JCM 16340^T^ (BAAAFQ000000000).

	dDDH* value (%)						
		**1**	**2**	**3**	**4**	**5**	**6**
**ANI** *value (%)	**1**	–	21.3	20.4	21.5	21.4	20.6
	**2**	71.3	–	22.1	21.6	21.3	20.7
	**3**	71.2	78.9	–	21.5	20.8	20.5
	**4**	70.2	70.6	70.6	–	19.8	20.2
	**5**	70.8	71.2	71.2	72.6	–	20.8
	**6**	70.6	71.3	70.8	72.7	77.3	–

†*ANI, average nucleotide identity; dDDH, digital DNA–DNA hybridization.

**Table 3.** Genome relatedness between strain G1-23^T^ and closely related type strains of the genus *Paraglaciecola* Strains: 1, G1-23^T^ (JAKGAS000000000); 2, *P. marina* D3211^T^ (SAIS00000000); 3, *P. aquimarina* KCTC 32108^T^ (JAWDIO000000000); 4, *P. psychrophila* 170^T^ (BAES00000000); 5, *P. arctica* BSs20135^T^ (BAEO00000000); 6, *P. hydrolytica* S66^T^ (LSNE00000000).

	dDDH* value (%)						
		**1**	**2**	**3**	**4**	**5**	**6**
**ANI** *value (%)	**1**	–	19.9	20.1	19.1	19.5	19.8
	**2**	72.4	–	20.1	19.9	20	19.1
	**3**	74.6	72.3	–	19.3	20.2	20.4
	**4**	72.5	72.8	72.1	–	24.6	19.5
	**5**	72.9	73.2	72.6	81.1	–	18.8
	**6**	70.9	70.5	70.5	71.2	71.4	–

†*ANI, average nucleotide identity; dDDH, digital DNA–DNA hybridization.

## Genomic features and algal symbiosis-associated genes

The genomes of strains G1-22^T^ and G1-23^T^, as well as their respective reference strains, were annotated using the NCBI Prokaryotic Genome Annotation Pipeline. A comparison of the genomes of strains G1-22^T^ and G1-23^T^ revealed similar general features, as summarized in Table 1. Most of the genomic features, including genome sizes, total gene numbers, protein-coding genes, and total tRNA gene numbers, exhibited similarities with their closely related reference strains of the genera *Psychrosphaera* and *Paraglaciecola*. Additionally, the DNA G+C contents of strains G1-22^T^ and G1-23^T^, calculated from their whole genome sequences, were 40.2 and 38.9 mol%, respectively, falling within the DNA G+C content ranges of the genera *Psychrosphaera* and *Paraglaciecola* [1,13].

Algae are primarily composed of polysaccharides, integral to their extracellular matrices, cell walls, and storage compounds. Consequently, the ability to degrade various algal polysaccharides is a significant trait among heterotrophic bacteria associated with marine algae [27]. Thus, we conducted a genome-wide analysis to investigate the distribution of genes encoding Carbohydrate Active enZymes (CAZy) in the genomes of strains G1-22^T^ and G1-23^T^, as well as closely related *Psychrosphaera* and *Paraglaciecola* species, using the dbCAN3 meta server (https://bcb.unl.edu/dbCAN2/blast.php) [28]. The genomes of strains G1-22^T^ and G1-23^T^ are predicted to contain a total of 166 and 199 genes encoding various CAZys, respectively. These numbers generally exceeded those observed in the reference strains of *Psychrosphaera* and *Paraglaciecola* (Table 1). This suggests that strains G1-22^T^ and G1-23^T^, isolated from marine algae, may possess enhanced capabilities to utilize cell-wall components from marine algae compared to other species of *Psychrosphaera* and *Paraglaciecola*. Notably, genes belonging to the glycoside hydrolase and glycosyltransferase families were abundantly identified among the six major CAZyme categories, indicating their potential to degrade a variety of red algal polysaccharides into simpler sugars, which can then be utilized as energy and carbon sources.

Symbiotic bacteria promote algal growth by providing plant hormones, vitamins, siderophores, and nutrients [29]. Genomic analysis revealed that strains G1-22^T^ and G1-23^T^ may harbour genes responsible for the synthesis of various B vitamins. Specifically, strain G1-23^T^ possesses the gene clusters *thiCDE* and *ribBEH* for the biosynthesis of thiamine (vitamin B_1_) and riboflavin (vitamin B_2_), respectively, suggesting that strain G1-23^T^ may have the ability to produce thiamine phosphate from 5-aminoimidazole ribotide and riboflavin from ribulose-5-phosphate. Additionally, strain G1-23^T^ contains the *cbiBP* and *cobASU* genes, which are involved in the biosynthesis of cobalamin (vitamin B_12_) from cobyrinate a,c-diamide [30]. In contrast, strain G1-22^T^ possesses only the genes *thiDE*, *ribEH*, *cbiP*, and *cobSU*, lacking the *thiC*, *ribB*, *cobA*, and *cbiB*, which encode phosphomethylpyrimidine synthase, 3,4-dihydroxy 2-butanone 4-phosphate synthase, cob(I)alamin adenosyltransferase, and adenosylcobinamide-phosphate synthase, respectively. These results suggest that strain G1-23^T^ may have the ability to synthesize vitamins B_1_, B_2_, and B_12_ from basic precursors, whereas strain G1-22^T^ may require intermediates, possibly derived from other organisms, to synthesize these vitamins. Both strains G1-22^T^ and G1-23^T^ possess genes for the biosynthesis of pantothenate (vitamin B_5_) from l-valine (*ilvE* and *panBCE*) and folate (vitamin B_9_) from GTP (*folABCEKP*, *phoD*), indicating their ability to synthesize these vitamins. Additionally, both strains harbour the gene encoding bacterioferritin (*bfr*) as a siderophore, which enhances iron availability and potentially promotes marine algal growth. Notably, strain G1-23^T^ possesses genes involved in phenylacetate synthesis (*katG* and *amiE*), a phytohormone that may enhance the growth and stress tolerance of marine algae [29]. The genomic analysis suggests that strains G1-22^T^ and G1-23^T^, particularly strain G1-23^T^, may contribute to the growth or survival of marine algae through the production of various beneficial compounds in algal sphere.

## Morphological and physiological characteristics

The growth ability of strains G1-22^T^ and G1-23^T^ on various standard bacteriological agar media (all sourced from MBcell), including MA, Reasoner’s 2A (R2A) agar, Luria-Bertani (LB) agar, tryptic soy agar (TSA), and nutrient agar (NA), was evaluated at 25 °C for 2 days. The NaCl concentrations in R2A agar, LB agar, TSA, and NA were adjusted to 2% (w/v). The growth of strains G1-22^T^ and G1-23^T^ was assessed at different temperatures (5–40 °C at 5 °C intervals) for 2 days on MA, and at different pH values (ranging from pH 4.0 to 10.0 at 1.0 pH unit intervals) in MB at 25 °C for 2 days. Sodium citrate, Na_2_HPO_4_/NaH_2_PO_4_, and sodium carbonate–bicarbonate buffers were used to prepare MB media with pH values of 4.0–5.0, 6.0–8.0, and 9.0–10.0, respectively. The pH levels were adjusted after autoclaving (at 121 °C for 15 min) if necessary. Salt tolerance was examined in MB with varying NaCl concentrations (ranging from 0 to 10% at 1.0% intervals, w/v) prepared in the laboratory according to the MB formula. Anaerobic growth was assessed on MA for 21 days at 25 °C under anaerobic conditions created using the GasPak Plus system (BBL).

Cell morphology, size, and the presence of flagella were examined using transmission electron microscopy (JEM-1010, jeol) and phase-contrast microscopy (Axio Scope.A1, Carl Zeiss,). The gliding motility of strains G1-22^T^ and G1-23^T^ was tested using MA containing 0.3% agar, following a previously described method [31]. Gram staining was performed using a Gram stain kit from bioMérieux, following the manufacturer’s instructions. Oxidase activity was determined by observing the oxidation of 1% (w/v) tetramethyl-*p*-phenylenediamine (Merck), while catalase activity was evaluated by the production of oxygen bubbles in a 3% (v/v) aqueous hydrogen peroxide solution [32]. The phenotypic characteristics of strains G1-22^T^ and G1-23^T^ were examined alongside reference strains under the same conditions at their respective optimal temperatures. Hydrolysis of casein (1% skimmed milk, w/v), starch (1%), aesculin (0.1%), l-tyrosine (0.5%), Tween 20(1%), and Tween 80(1%) was assessed on MA following established protocols [33]. Additional biochemical features and enzymatic activities of strains G1-22^T^ and G1-23^T^ and their reference strains were analysed using the API 20NE and API ZYM kits from bioMérieux, following the manufacturer’s instructions, with adjustments made to the solutions in the API kits to approximately 2% NaCl.

Strains G1-22^T^ and G1-23^T^ exhibited robust growth on MA but did not grow on TSA, NA, LB agar, and R2A agar containing 2% NaCl. Cells of strain G1-22^T^ were Gram-stain-negative, non-motile rods, measuring 0.5–0.6×1.4–1.7 µm, while cells of strain G1-23^T^ were Gram-stain-negative, non-motile rods, measuring 0.9–1.1×2.0–2.5 µm (Fig. S2). Anaerobic growth was not observed for strains G1-22^T^ and G1-23^T^ over 21 days, indicating their strict aerobic nature. Although strains G1-22^T^ and G1-23^T^, along with their closely related respective reference strains, shared several phenotypic features (e.g., oxidase and catalase activities, hydrolysis of Tween 20, Tween 80, and aesculin were positive while nitrate reduction, and indole production were negative for strain G1-22^T^; oxidase and catalase activities, hydrolysis of aesculin were positive while nitrate reduction, and hydrolysis of l-tyrosine, and Tween 20 were negative for strain G1-23^T^), there were also noticeable differences (e.g., colony colour, flagellum motility, growth conditions, and negative casein hydrolysis for strain G1-22^T^; colony colour, flagellum motility, growth conditions, negative casein hydrolysis and positive Tween 80 hydrolysis for strain G1-23^T^). These distinctions allow for the differentiation of strains G1-22^T^ and G1-23^T^ from their respective reference strains (Tables4  5). Significantly, the lack of flagellum motility in strains G1-22^T^ and G1-23^T^ serves as a clear distinguishing characteristic from other species within the genera *Psychrosphaera* and *Paraglaciecola*.

**Table 4.** Differential phenotypic characteristics of the strain G1-22^T^ and closely related type strains of the genus *Psychrosphaera* Strains: 1, G1-22^T^ (this study); 2, *Psychrosphaera saromensis* KCTC 23240^T^ [1]; 3, *Psychrosphaera aquimarina* KCTC 52743^T^ [3]; 4, *Psychrosphaera ytuae* JCM 34321^T^ [4]; 5, *Psychrosphaera aestuarii* PSC101^T^ [2]; 6, *Psychrosphaera haliotis* KDW4^T^ [2]. All strains are positive for the following characteristics: activity* of catalase, oxidase, alkaline phosphatase, esterase (C4), leucine arylamidase, hydrolysis* of Tween 20, Tween 80. All strains are negative for the following characteristics: nitrate reduction, indole production, glucose fermentation, activity* of *β*-glucuronidase, *α-*mannosidase, *α*-fucosidase, *α*-galactosidase, *α*-glucosidase, and *β*-glucosidase, and assimilation* of l-arabinose, d-mannose, *N*-acetyl-glucosamine, capric acid, malic acid, trisodium citrate, and phenylacetic acid. +, Positive; –, negative; na, not available.

Characteristic	1	2	3	4	5	6
Isolation source	Marine alga	Lake water	Seawater	Sea sediment	Seawater	Marine invertebrate
Pigment	Cream	None	White	None	None	None
Flagella motility	−	+	+	+	+	+
Range for growth:						
Temperature (°C)	15–30	4–30	10–30	4–40	4–37	4–37
pH	7.0–9.0	6.0–9.0	6.0–9.0	6.0–10.0	6.0–9.0	6.0–9.0
NaCl (%)	1–7	1–5	1–5	1–11	0.5–10	1–12
Activity* of:						
Arginine dihydrolase	+	+	+	+	na	na
Esterase lipase (C8), acid phosphatase	+	+	+	+	−	+
Lipase (14), trypsin, *α*-chymotrypsin, naphthol-AS-BI-phosphohydrolase, urease	+	+	+	+	−	−
*β*-Galactosidase	+	−	−	−	−	−
*N*-Acetyl-*β*-glucosaminidase	+	−	+	−	−	−
Hydrolysis* of:						
Casein	−	+	−	+	+	+
Gelatin	−	−	−	−	+	−
Aesculin	+	+	+	+	−	−
Starch, l-tyrosine	−	−	−	−	+	+
Assimilation* of:						
d-Glucose	+	−	+	−	−	−
d-Mannitol, adipic acid, maltose	+	+	−	+	−	−
Potassium gluconate	−	−	+	+	−	−

*All data, except for those pertaining to *P. aestuarii* and *P. haliotis*, were obtained from this study. Data for *P. aestuarii* and *P. haliotis* were obtained from their reference source [2].

**Table 5.** Differential phenotypic characteristics of strain G1-23^T^ and closely related type strains of the genus *Paraglaciecola* Strains: 1, G1-23^T^ (this study); 2, *Paraglaciecola marina* KCTC 72122^T^ [6]; 3, *Paraglaciecola aquimarina* KCTC 32108^T^ [8]; 4, *Paraglaciecola psychrophila* 170^T^ [10]; 5, *Paraglaciecola arctica* BSs20135^T^ [11]; 6, *Paraglaciecola hydrolytica* S66^T^ [12]. All strains are positive for the following characteristics: activity* of catalase, oxidase, alkaline phosphatase, leucine arylamidase, and naphthol-AS-BI-phosphohydrolase. All strains are negative for the following characteristics: nitrate reduction, indole production, and assimilation* of phenylacetic acid. Symbols: +, positive; –, negative; na, not available.

Characteristic	1	2	3	4	5	6
Isolation source	Marine alga	Marine alga	Seawater	Arctic	Arctic	Marine alga
Pigment	Cream	Yellow	Greyish yellow	Nonn	Yellowish brown	Beige to pale orange
Flagella motility	−	+	+	+	+	+
Range for growth:						
Temperature (°C)	15–30	4–45	4–37	4–15	4–28	10–25
pH	7.0–10.0	6.0–9.0	7.0–8.0	6.0–9.0	na	7.0–9.0
NaCl (%)	1–7	1–9	0.5–5	1–6	1–5	1–6
Glucose fermentation*	−	−	−	−	+	+
Activity* of:						
Arginine dihydrolase, urease	+	+	+	−	−	−
Esterase (C4), esterase lipase (C8), acid phosphatase	+	+	+	−	+	+
Trypsin	−	−	−	+	+	+
*α*-Chymotrypsin	−	−	−	+	−	+
*β*-Glucuronidase, *α*-mannosidase, *α*-fucosidase	−	−	−	−	−	na
Lipase (C14)	+	−	+	+	−	na
*β*-Galactosidase	+	−	+	+	−	+
*β*-Glucosidase, *N*-acetyl-*β*-glucosaminidase	+	−	+	−	−	+
*α*-Galactosidase, *α*-glucosidase	−	−	+	−	−	na
Hydrolysis* of:						
Aesculin	+	+	+	−	+	+
Gelatin	−	+	−	−	−	−
Starch	−	+	−	+	−	+
Casein	−	+	+	−	−	na
Tween 80	+	−	−	+	−	na
l-Tyrosine, Tween 20	−	−	−	na	na	na
Assimilation* of:						
Adipic acid	+	+	+	−	−	na
l-Arabinose	−	−	+	−	−	+
d-Mannose	+	+	−	−	−	+
*N*-Acetyl-glucosamine	+	+	−	−	−	−
d-Mannitol	+	−	+	−	−	+
Potassium gluconate	−	+	+	+	−	na
d-Glucose	−	−	−	−	−	+
Maltose	−	−	−	+	−	+
Capric acid	−	−	−	−	na	−
Malic acid	−	−	−	−	−	na
Trisodium citrate	−	−	−	−	−	−

*All data, except for those pertaining to *P. psychrophila*, *P. arctica*, and *P. hydrolytica*, were obtained from this study. Data for *P. psychrophila*, *P. arctica*, and *P. hydrolytica* were obtained from their reference sources [10,12].

## Chemotaxonomic characteristics

The respiratory isoprenoid quinones of strains G1-22^T^ and G1-23^T^ were extracted following the method outlined by Minnikin *et al*. [34], and subsequently analysed using an HPLC system (LC-20A, Shimadzu) equipped with a reversed-phase Kromasil column (250×4.6 mm; Akzo Nobel Center) and a diode array detector (SPD-M20A, Shimadzu). Methanol-isopropanol (2 : 1, v/v) served as the eluent solution at a flow rate of 1 ml min^−1^. For the analysis of cellular fatty acids, strains G1-22^T^ and G1-23^T^ were aerobically cultivated in MB at their optimal temperatures alongside reference strains and harvested during the exponential growth phase (optical density, OD_600_=0.7–0.8). The cellular fatty acids were extracted from bacterial cells in accordance with the standard midi protocol, involving saponification, methylation, and extraction. The resulting fatty acid methyl esters were then analysed using a 6890-gas chromatograph (Hewlett Packard) and identified utilizing the RTSBA6 database of the Microbial Identification System (Sherlock version 6.0B) [35]. The polar lipid compositions of strains G1-22^T^ and G1-23^T^ were analysed using two-dimensional TLC, following the procedure outlined by Minnikin *et al*. [36]. Different types of polar lipids were identified using the following reagents: 10% ethanolic molybdophosphoric acid (for total polar lipids), ninhydrin (for aminolipids), Dittmer–Lester reagent (for phospholipids), and *α*-naphthol/sulphuric acid (for glycolipids). The presence of PE, PG, and DPG in strains G1-22^T^ and G1-23^T^ was confirmed utilizing standard polar lipid compounds procured from Sigma-Aldrich.

Strains G1-22^T^ and G1-23^T^ were found to contain Q-8 as the sole respiratory isoprenoid quinone, consistent with other species of the genera *Psychrosphaera* [1,4] and *Paraglaciecola* [5,13]. As for major cellular fatty acids (> 5% of the total fatty acids), strain G1-22^T^ included C_16 : 0_, summed feature 3 (C_16 : 1_ *ω*7*c* and/or C_16 : 1_ *ω*6*c*), summed feature 8 (C_18 : 1_ *ω*7*c* and/or C_18 : 1_ *ω*6*c*), C_12 : 1_ 3-OH, and C_10 : 0_ 3-OH (Table S1), while strain G1-23^T^ contained C_16 : 0_, summed feature 3 (C_16 : 1_ *ω*7*c* and/or C_16 : 1_ *ω*6*c*), and C_14 : 0_ (Table S2). Although the overall fatty acid profiles of strains G1-22^T^ and G1-23^T^ closely resembled those of their closely related reference strains of the genera *Psychrosphaera* and *Paraglaciecola*, there were some differences in the proportions of specific fatty acids (Tables S1 and S2). PE, PG, and DPG were identified as major polar lipids in both strains G1-22^T^ and G1-23^T^. Additionally, two unidentified polar lipids were detected in strain G1-22^T^, while an unidentified polar lipid and an unidentified aminophospholipid were detected in strain G1-23^T^ (Fig. S3). These polar lipid profiles were similar to those observed in *Psychrosphaera* [2,4] and *Paraglaciecola* [6,9] species.

## Taxonomic conclusion

The combined evidence from phylogenetic analysis, along with physiological and chemotaxonomic traits, strongly suggests that strains G1-22^T^ and G1-23^T^ belong to two distinct novel species within the genera *Psychrosphaera* and *Paraglaciecola*, respectively. Therefore, we propose the names *Psychrosphaera algicola* sp. nov. and *Paraglaciecola algarum* sp. nov. for these newly identified species, respectively.

## Reclassification of *Pseudoalteromonas elyakovii*, *Pseudoalteromonas flavipulchra*, and *Pseudoalteromonas profundi* as later heterotypic synonyms of *Pseudoalteromonas distincta*, *Pseudoalteromonas maricaloris*, and *Pseudoalteromonas gelatinilytica*

The phylogenetic trees reconstructed based on 16S rRNA gene sequences using NJ, ML, and MP algorithms, as well as the genome-based phylogenetic tree, unequivocally demonstrated that *Pseudoalteromonas elyakovii* and *Pseudoalteromonas distincta* [37,40], *Pseudoalteromonas flavipulchra* and *Pseudoalteromonas maricaloris* [41], and *Pseudoalteromonas profundi* and *Pseudoalteromonas gelatinilytica* [42,43], previously identified as distinct species, each formed tight phylogenetic lineages within the genus *Pseudoalteromonas* (Figs1^ 3^ and S1). The 16S rRNA gene sequence similarities between their respective type strains were 100%, 99.9%, and 99.2%, respectively. Moreover, ANI and dDDH values between the genomes of *P. distincta* ATCC 700518^T^ (JWIG00000000) and *P. elyakovii* LMG 14908^T^ (JASGWX000000000), *P. flavipulchra* LMG 20361^T^ (VSSD00000000) and *P. maricaloris* LMG 19692^T^ (WEIA00000000), and *P. profundi* CGMCC 1.15394^T^ (BMIT00000000) and *P. gelatinilytica* NH153^T^ (LRRU00000000) were found to be 100 and 100%, 98.8 and 89.6%, and 97.7 and 79.0% (Table S3), respectively, surpassing the thresholds established for prokaryotic species delineation [33]. These findings strongly indicate that *P. distincta* and *P. elyakovii*, *P. flavipulchra* and *P. maricaloris*, and *P. profundi* and *P. gelatinilytica* should be considered conspecific species pairs. Consequently, we propose the reclassification of *Pseudoalteromonas elyakovii* (Ivanova *et al*. 1987) Sawabe *et al*. 2000, *Pseudoalteromonas flavipulchra* Ivanova *et al*. 2002, and *Pseudoalteromonas profundi* Zhang *et al*. 2016 should be reclassified as later heterotypic synonyms of *Pseudoalteromonas distincta* (Romanenko *et al*. 1995) Ivanova *et al*. 2000, *Pseudoalteromonas maricaloris* Ivanova *et al*. 2002, and *Pseudoalteromonas gelatinilytica* Yan *et al*. 2016, respectively, in this study.

## Description of *Psychrosphaera algicola* sp. nov.

*Psychrosphaera algicola* (al.gi′co.la. L. fem. n. *alga*, an alga; L. suffix. -*cola*, (from L. masc. or fem. n. *incola*) inhabitant, dweller; N.L. fem. n. *algicola*, an alga dweller).

Colonies grown on MA display a smooth, circular, slightly convex shape with a creamy colour. Cells are Gram-stain-negative, obligately aerobic, and non-motile rods, but gliding motility is positive. Growth occurs at 15–30 °C (optimum, 25–30 °C) and pH 7.0–9.0 (optimum, pH 8.0) and in the presence of 1.0–7.0% (w/v) NaCl (optimum, 2.0–3.0%). Oxidase- and catalase-positive. Nitrate is not reduced to nitrite. Indole production and d-glucose fermentation are negative. Hydrolysis of aesculin, Tween 20, and Tween 80 is positive, but hydrolysis of l-tyrosine, casein, starch, and gelatin is negative. Arginine dihydrolase, alkaline phosphatase, esterase (C4), esterase lipase (C8), lipase (C14), trypsin, *α*-chymotrypsin, leucine arylamidase, acid phosphatase, naphthol-AS-BI-phosphohydrolase, *β*-galactosidase, and *N-*acetyl-*β*-glucosaminidase, and urease activities are positive, but *β*-glucuronidase, *α-*mannosidase, *α-*fucosidase, *α-*galactosidase, *α*-glucosidase, and *β*-glucosidase activities are negative. Assimilation of d-glucose, d-mannitol, maltose, and adipic acid is positive, but assimilation of potassium gluconate, l-arabinose, d-mannose, *N-*acetyl-glucosamine, capric acid, malic acid, trisodium citrate, and phenylacetic acid is negative. Q-8 is the sole respiratory quinone. The major cellular fatty acids (>5%) are C_16 : 0_, summed feature 3 (C_16 : 1_ *ω*7*c* and/or C_16 : 1_ *ω*6*c*), summed feature 8 (C_18 : 1_ *ω*7*c* and/or C_18 : 1_ *ω*6*c*), C_12 : 1_ 3-OH, and C_10 : 0_ 3-OH. PE, PG, DPG, and two unidentified lipids are identified as major polar lipids.

The type strain is G1-22^T^ (=KACC 22486^T^=JCM 34971^T^), isolated from the phycosphere of a *Sargassum* species, a marine brown alga collected in Gangwon province, Republic of Korea. The genome size of the type strain is 4695 kb, with a DNA G+C content of 40.2 mol% (calculated from the whole genome sequence). The GenBank accession numbers for the 16S rRNA gene and genome sequences of strain G1-22^T^ are OK398154 and JAQOMS000000000, respectively.

## Description of *Paraglaciecola algarum* sp. nov.

*Paraglaciecola algarum* (al.ga'rum. L. gen. pl. n. *algarum*, of/from algae).

Colonies grown on MA display a smooth, circular, slightly convex shape with a creamy colour. Cells are Gram-stain-negative, obligately aerobic, and non-motile rods, but gliding motility is positive. Growth occurs at 15–30 °C (optimum, 25–30 °C) and pH 7.0–10.0 (optimum, pH 8.0) and in the presence of 1.0–7.0% (w/v) NaCl (optimum, 4.0%). Oxidase- and catalase-positive. Nitrate is not reduced to nitrite. Indole production and d-glucose fermentation are negative. Hydrolysis of aesculin and Tween 80 is positive, but hydrolysis of l-tyrosine, casein, starch, gelatin, and Tween 20 is negative. Arginine dihydrolase, alkaline phosphatase, esterase (C4), esterase lipase (C8), lipase (C14), leucine arylamidase, acid phosphatase, naphthol-AS-BI-phosphohydrolase, *β-*galactosidase, *β*-glucosidase, *N-*acetyl-*β*-glucosaminidase, and urease activities are positive, but trypsin, *α*-chymotrypsin, *β-*glucuronidase, *α*-mannosidase, *α*-fucosidase, *α-*galactosidase, and *α-*glucosidase activities are negative. Assimilation of adipic acid, d-mannose, d-mannitol, and *N*-acetyl-glucosamine is positive, but assimilation of d-glucose, maltose, l-arabinose, potassium gluconate, capric acid, malic acid, trisodium citrate, and phenylacetic acid is negative. Q-8 is the sole respiratory quinone. The major cellular fatty acids (>5%) are C_16 : 0_, summed feature 3 (C_16 : 1_ *ω*7*c* and/or C_16 : 1_ *ω*6*c*), and C_14 : 0_. PE, PG, DPG, an unidentified aminophospholipid, and an unidentified lipid are identified as the major polar lipids.

The type strain is G1-23^T^ (=KACC 22490^T^=JCM 34972^T^), isolated from the phycosphere of a *Sargassum* species, a marine brown alga collected in Gangwon province, Republic of Korea. The genome size of the type strain is 4818 kb, with a DNA G+C content of 38.9 mol% (calculated from the whole genome sequence). The GenBank accession numbers for the 16S rRNA gene and genome sequences of strain G1-23^T^ are OK377038 and JAKGAS000000000, respectively.

## Emended description of *Pseudoalteromonas distincta*

Heterotypic synonym: *Pseudoalteromonas elyakovii* (Ivanova *et al*. 1997) Sawabe *et al*. 2000.

The description is as given for *Alteromonas distincta* [39] and *Pseudoalteromonas distincta* [38] with the following modifications. The DNA G+C contents of the genomic DNA range from 38.5 to 39.3 mol%. The type strain is KMM 638^T^ (=ATCC 700518^T^=DSM 12749^T^). The GenBank accession numbers for the 16S rRNA gene and genome sequences of the type strain are AF082564 and JWIG00000000, respectively.

## Emended description of *Pseudoalteromonas maricaloris*

Heterotypic synonym: *Pseudoalteromonas flavipulchra* Ivanova *et al*. 2002.

The description is as given for *Pseudoalteromonas maricaloris* [41] with the following modifications. The DNA G+C contents of the genomic DNA range from 38.9 to 41.7 mol%. The type strain is KMM 636^T^ (=LMG 19692^T^=CIP 106859^T^). The GenBank accession numbers for the 16S rRNA gene and genome sequences of the type strain are AF144036 and WEIA00000000, respectively.

## Emended description of *Pseudoalteromonas gelatinilytica*

Heterotypic synonym: *Pseudoalteromonas profundi* Zhang *et al*. 2016.

The description is as given for *Pseudoalteromonas gelatinilytica* [43] with the following modifications. The DNA G+C contents of the genomic DNA range from 41.4 to 46.7 mol%. The type strain is NH153^T^ (=CGMCC 1.15370^T^=DSM 100951^T^). The GenBank accession numbers for the 16S rRNA gene and genome sequences of the type strain are KT377064 and LRRU00000000, respectively.

## supplementary material

10.1099/ijsem.0.006491 Uncited Supplementary Material 1.

## Abbreviations

ANI: average nucleotide identity
ASW: artificial seawater
CAZy: carbohydrate active enZyme
dDDH: digital DNA–DNA hybridization
DPG: diphosphatidylglycerol
MA: marine agar
MB: marine broth
ML: maximum-likelihood
MP: maximum-parsimony
NJ: neighbour-joining
PE: phosphatidylethanolamine
PG: phosphatidylglycerol
Q-8: ubiquinone-8
