# Supplementary material for: Psychrosphaera algicola sp. nov. and Paraglaciecola algarum sp. nov., and reclassification of Pseudoalteromonas elyakovii, Pseudoalteromonas flavipulchra, and Pseudoalteromonas profundi as later heterotypic synonyms of P. distincta, P. maricaloris, and P. gelatinilytica
Source: Int J Syst Evol Microbiol. 2024 Aug 14;74(8):006491. doi: 10.1099/ijsem.0.006491 (PMC11324256; doi:10.1099/ijsem.0.006491)
Supplement: Uncited Supplementary Material 1. [file ijsem-74-06491-s001.pdf]

Supplementary Information

Psychrosphaera algicola sp. nov. and *Paraglaciecola algarum* sp. nov., and reclassification of *Pseudoalteromonas elyakovii*, *Pseudoalteromonas flavipulchra*, and *Pseudoalteromonas profundi* as later heterotypic synonyms of *P. distincta*, *P. maricaloris*, and *P. gelatinilytica*

Hülya Bayburt[†], Byeong Jun Choi[†], Jeong Min Kim, Ju Hye Baek, and Che Ok Jeon[*]
Department of Life Science, Chung-Ang University, Seoul 06974, Republic of Korea

*Corresponding author [Che Ok Jeon]: Department of Life Science, Chung-Ang University, 84, HeukSeok-Ro, Dongjak-Gu, Seoul 06974, Republic of Korea.
Tel: +82-2-820-5864, E-mail: cojeon@cau.ac.kr
[†]These authors contributed equally to this work.

Fig. S1. Maximum-likelihood (ML; a, c) and maximum-parsimony (MP; b, d) trees based on 16S rRNA gene sequences showing the phylogenetic relationships between strains G1-22^{T} (a, b) and G1-23^{T} (c, d) and their closely related taxa. Bootstrap values greater than 70% based on 1000 replicates are indicated. *Idiomarina abyssalis* KM227(EI4)^{T} (AF052740) and *Cognaticolwellia beringensis* NB097-1^{T} (MG016490) were employed as the outgroups for strains G1-22^{T} and G1-23^{T}, respectively. The scale bars in the ML and MP trees indicate nucleotide changes per nucleotide positions and nucleotide changes over the whole 16S rRNA sequences, respectively.

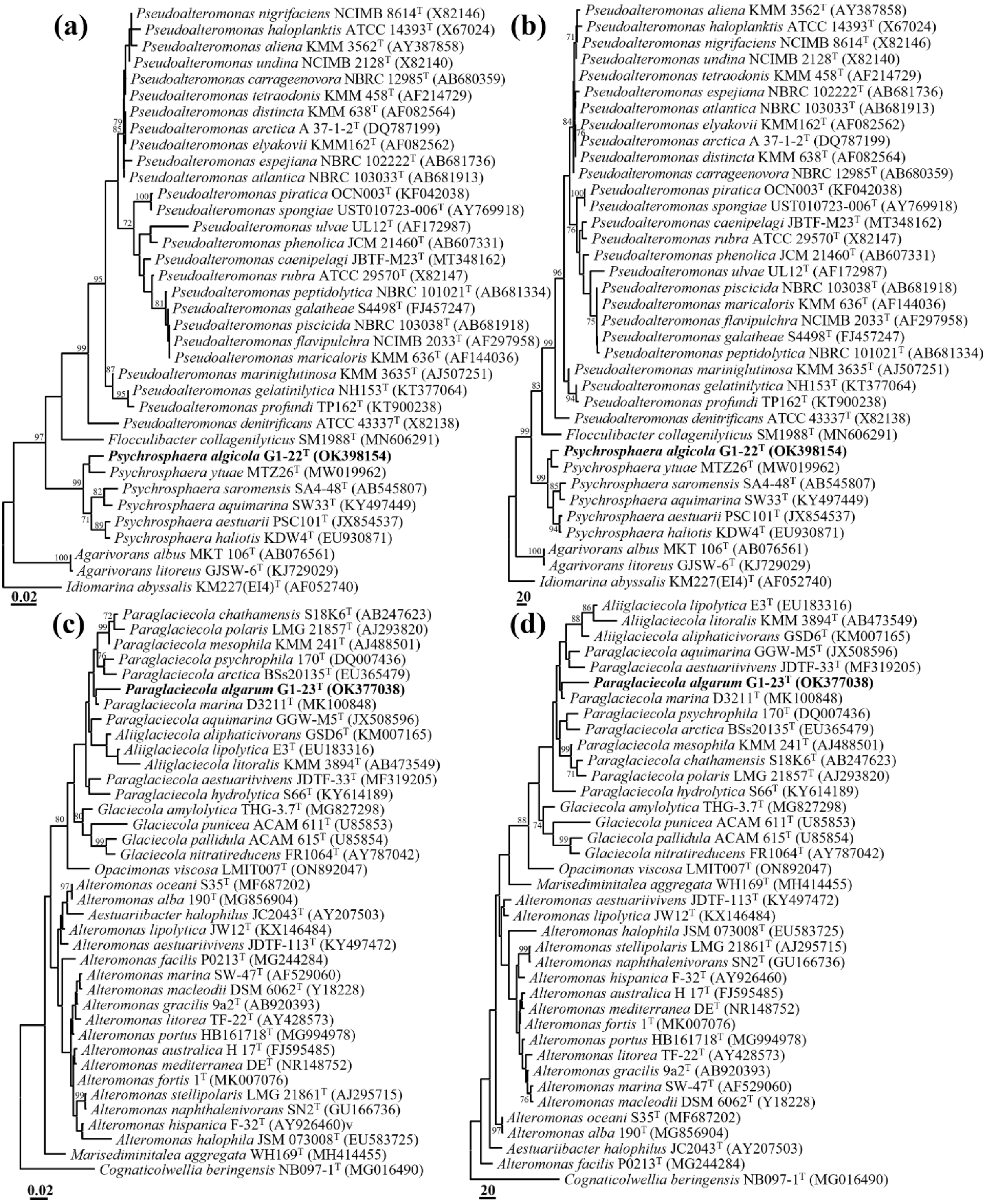

Fig. S2. Transmission electron micrographs of negatively stained (using 2% (w/v) uranyl acetate) cells showing the general morphologies of strains G1-22^{T} (a) and G1-23^{T} (b) grown on marine agar for 2 days. Scale bars: 1 μm.

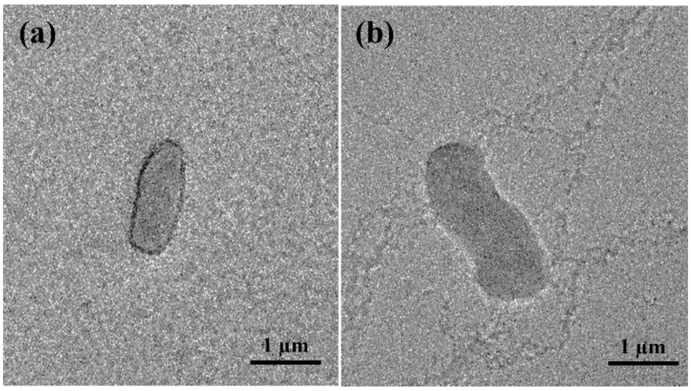

Fig. S3. Two-dimensional thin-layer chromatograms (TLC) showing the polar lipid profiles of strains G1-22^{T} and G1-23^{T}. Solvent systems: (I) chloroform-methanol-water (65:25:4, v/v/v) and (II) chloroform-acetic acid-methanol-water (80:15:12:4, v/v/v/v). The TLC plates were sprayed with 10% ethanolic molybdophosphoric acid (A), ninhydrin (B), Dittmer-Lester (C), and α-naphthol/sulfuric acid (D) reagents for the detection of total polar lipids, aminolipids, phospholipids, and glycolipids, respectively. PG, phosphatidylglycerol; DPG, diphosphatidylglycerol; PE, phosphatidylethanolamine; APL, unidentified aminophospholipid; PL, unidentified phospholipid.

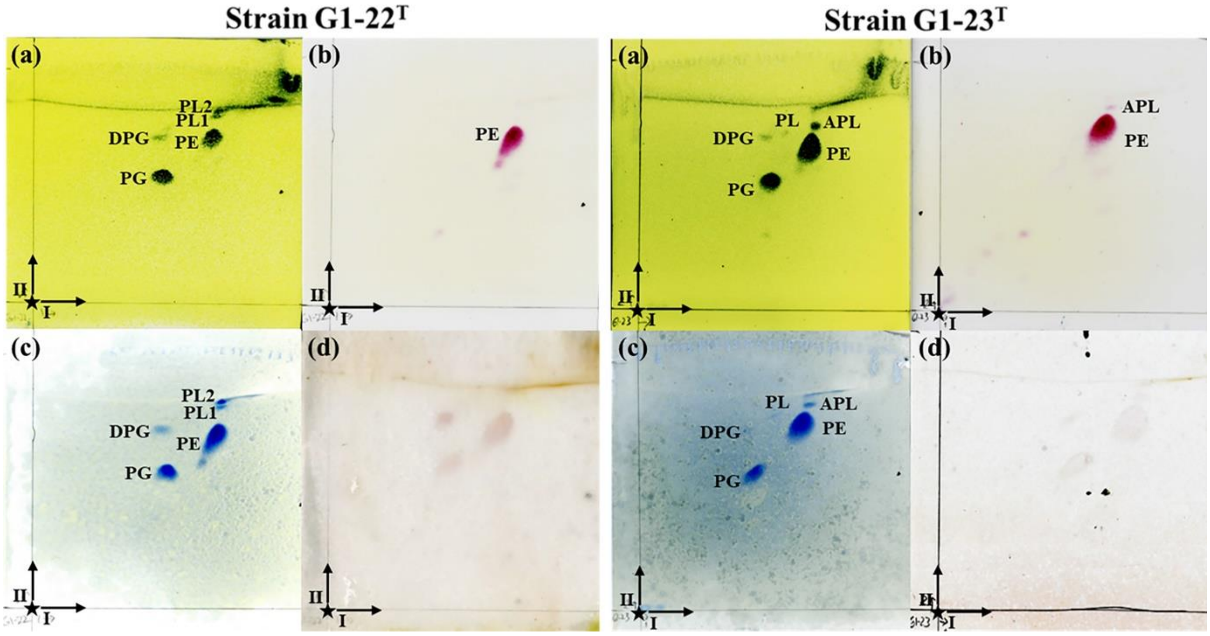

Table S1. Cellular fatty acid compositions (%) of strain G1-22^{T} and closely related type strains of the genus *Psychrosphaera*

Taxa: 1, strain G1-22^{T}; 2, *P. saromensis* KCTC 23240^{T}; 3, *P. aquimarina* KCTC 52743^{T}; 4, *P. ytuae* JCM 34321^{T}. All data were obtained from this study. Data are expressed as percentages of the total fatty acids and fatty acids amounting to less than 1.0% in all strains are not shown. Major components (> 5.0%) are highlighted in bold. Symbols: tr, trace amount (< 1.0%); –, not detected.

**Fatty acid**	**1**	**2**	**3**	**4**
Saturated:				
$C_{11:0}$	–	–	1.1	tr
$C_{12:0}$	2.2	tr	1.2	tr
$C_{13:0}$	tr	tr	2.1	1.2
$C_{14:0}$	2.7	1.2	2.3	2.0
$C_{16:0}$	**29.1**	**26.0**	**22.2**	**11.1**
$C_{17:0}$	2.7	**5.6**	**8.9**	**6.5**
$C_{18:0}$	1.20	tr	tr	Tr
Unsaturated:				
$C_{15:1}$ *ω8c*	2.5	**6.1**	**17.5**	**6.3**
$C_{17:1}$ *ω6c*	–	tr	1.04	tr
$C_{17:1}$ *ω8c*	3.6	**5.0**	**5.1**	**18.0**
$C_{20:1}$ *ω7c*	1.1	tr	tr	tr
Hydroxy:				
$C_{9:0}$ 3-OH	–	tr	1.09	–
$C_{10:0}$ 3-OH	**6.9**	3.2	3.7	**5.4**
$C_{11:0}$ 3-OH	–	1.1	tr	3.9
$C_{12:1}$ 3-OH	**9.7**	4.8	**6.5**	tr
$C_{16:0}$ 3-OH	tr	tr	–	1.1
Branched:				
iso-$C_{16:0}$	tr	tr	2.0	tr
Summed features*:				
3	**23.4**	**34.9**	**16.1**	**16.5**
7	–	tr	tr	1.6
8	**9.0**	**6.3**	2.8	**15.7**

*Summed features are fatty acids that cannot be resolved reliably from another fatty acid using the chromatographic conditions chosen. The MIDI system groups these fatty acids together as one feature with a single percentage of the total. Summed feature 3, $C_{16:1}$ *ω7c* and/or $C_{16:1}$ *ω6c*; summed feature 7, $C_{19:1}$ *ω7c* and/or $C_{19:1}$ *ω6c*; summed feature 8, $C_{18:1}$ *ω7c* and/or $C_{18:1}$ *ω6c*.

Table S2. Cellular fatty acid compositions (%) of strain G1-23^T and closely related type strains of the genus *Paraglaciecola*

Taxa: 1, strain G1-23^T; 2, *P.. aquimarina* KCTC 32108^T; 3, *P. marina* KCTC 72122^T. All data were obtained from this study. Data are expressed as percentages of the total fatty acids and fatty acids amounting to less than 1.0% in all strains are not shown. Major components (> 5.0%) are highlighted in bold. Symbols: tr, trace amount (< 1.0%); –, not detected.

**Fatty acid**	**1**	**2**	**3**
Saturated:			
$C_{12:0}$	1.2	3.4	2.0
$C_{14:0}$	**6.0**	**5.6**	1.5
$C_{16:0}$	**18.2**	**12.1**	**15.0**
$C_{18:0}$	tr	–	1.8
Unsaturated:			
$C_{15:1}$ *ω8c*	4.4	–	tr
$C_{16:1}$ *ω5c*	tr	–	1.3
$C_{17:1}$ *ω8c*	2.9	–	2.3
Hydroxy:			
$C_{10:0}$ 3-OH	1.8	tr	–
$C_{12:0}$ 3-OH	tr	**6.7**	1.4
$C_{12:1}$ 3-OH	3.0	3.9	**8.2**
$C_{13:0}$ 2-OH	–	tr	2.8
$C_{14:0}$ 2-OH	tr	**43.5**	–
$C_{17:0}$ 2-OH	–	–	1.3
iso-$C_{17:0}$ 3-OH	tr	–	1.2
Branched:			
iso-$C_{13:0}$	–	–	2.4
iso-$C_{15:0}$	tr	tr	3.3
iso-$C_{17:0}$	–	–	**7.8**
Summed features*:			
3	**52.5**	**14.7**	**23.4**
8	3.6	2.0	**5.8**
9	–	–	2.5

*Summed features are fatty acids that cannot be resolved reliably from another fatty acid using the chromatographic conditions chosen. The MIDI system groups these fatty acids together as one feature with a single percentage of the total. Summed feature 3, $C_{16:1}$ *ω7c* and/or $C_{16:1}$ *ω6c*; summed feature 8, $C_{18:1}$ *ω7c* and/or $C_{18:1}$ *ω6c*; summed feature 9, 10-methyl-$C_{16:0}$.

Table S3. Genome-relatedness analysis of type strains within the genus *Pseudoalteromonas*, showing that *P. distincta* and *P. elyakovii*, *P. flavipulchra* and *P. maricaloris*, *P. profundi* and *P. gelatinilytica* are conspecific species pairs.

Taxa: 1, *P. agarivorans* DSM 14585^{T} (CP011011); 2, *P. arctica* A 37-1-2^{T} (CP011025); 3, *P. carrageenovora* IAM 12662^{T} (AQGW00000000); 4, *P. distincta* ATCC 700518^{T} (JWIG00000000); 5, *P. elyakovii* LMG 14908^{T} (JASGWX000000000); 6, *P. espejiana* DSM 9414^{T} (CP011028); 7, *P. tetraodonis* GFC^{T} (CP011041); 8, *P. flavipulchra* LMG 20361^{T} (VSSD00000000); 9, *P. maricaloris* LMG 19692^{T} (WEIA00000000); 10, *P. profundi* CGMCC 1.15394^{T} (BMIT00000000); 11, *P. gelatinilytica* NH153^{T} (LRRU00000000); 12, *P. mariniglutinosa* NCIMB 1770^{T} (AQHC00000000); 13, *P. haloplanktis* CIP 103197^{T} (CAMAPB000000000).

		dDDH† value (%)												
		**1**	**2**	**3**	**4**	**5**	**6**	**7**	**8**	**9**	**10**	**11**	**12**	**13**
	**1**	–	24.1	28.1	24.6	24.5	31.4	22.9	21.2	22.3	20.5	20.7	21.2	23.3
	**2**	80.8	–	26.7	53.0	52.9	24.4	22.6	20.5	21.2	20.2	20.4	21.0	23.0
	**3**	83.9	83.5	–	27.5	27.4	30.8	22.6	19.8	20.4	20.1	20.2	20.5	22.8
	**4**	81.4	93.6	84.0	–	**100.0**	24.5	22.7	20.2	21.1	20.2	20.3	21.1	22.7
	**5**	81.3	93.6	84.0	**100.0**	–	24.5	22.6	19.9	20.9	20.2	20.2	21.0	22.6
ANI†	**6**	86.3	81.3	85.9	81.7	81.6	–	22.6	20.8	20.7	20.3	20.5	20.9	22.6
value	**7**	79.0	79.2	79.2	79.4	79.5	79.1	–	21.2	23.8	20.9	21.1	21.5	67.1
(%)	**8**	70.8	70.8	70.8	70.8	70.8	70.8	70.8	–	**89.6**	19.8	20.0	19.8	20.2
	**9**	70.8	70.8	70.9	70.8	71.0	70.8	71.3	**98.8**	–	21.0	21.3	21.0	23.0
	**10**	75.0	74.9	74.9	75.0	75.1	75.1	75.2	71.0	71.6	–	**79.0**	20.5	20.9
	**11**	75.0	74.9	75.0	75.0	75.2	75.1	75.3	71.2	71.6	**97.7**	–	20.6	21.0
	**12**	75.8	75.9	75.7	75.9	76.1	75.8	76.5	70.9	71.0	75.8	75.8	–	21.8
	**13**	79.3	79.3	79.1	79.5	79.4	79.0	96.1	71.0	71.5	75.4	75.5	76.8	–

†ANI, average nucleotide identity; dDDH, digital DNA-DNA hybridization.
